# Stochastic Assessment of the Economic Impact of *Streptococcus suis*-Associated Disease in German, Dutch and Spanish Swine Farms

**DOI:** 10.3389/fvets.2021.676002

**Published:** 2021-08-19

**Authors:** Carlos Neila-Ibáñez, Jordi Casal, Isabel Hennig-Pauka, Norbert Stockhofe-Zurwieden, Marcelo Gottschalk, Lourdes Migura-García, Lola Pailler-García, Sebastián Napp

**Affiliations:** ^1^Centre de Recerca en Sanitat Animal (CReSA), Institut de Recerca i Tecnologia Agroalimentàries (IRTA), Universitat Autònoma de Barcelona (UAB), Bellaterra, Spain; ^2^Office International des Epizooties Collaborating Centre for the Research and Control of Emerging and Re-emerging Diseases in Europe, Centre de Recerca en Sanitat Animal (CReSA), Institut de Recerca i Tecnologia Agroalimentàries (IRTA), Bellaterra, Spain; ^3^Department of Animal Health and Anatomy, Faculty of Veterinary Medicine, Universitat Autònoma de Barcelona, Bellaterra, Spain; ^4^Field Station for Epidemiology, University of Veterinary Medicine Hannover, Foundation, Hannover, Germany; ^5^Wageningen Bioveterinary Research, Wageningen University and Research, Lelystad, Netherlands; ^6^Research Group on Infectious Diseases in Production Animals and Swine and Poultry Infectious Diseases Research Centre, Faculty of Veterinary Medicine, University of Montreal, Saint-Hyacinthe, QC, Canada

**Keywords:** *Streptococcus suis*, antimicrobials, questionnaires, economic assessment, swine production disease, incidence, stochastic model, cost of disease

## Abstract

The economic assessment of animal diseases is essential for decision-making, including the allocation of resources for disease control. However, that assessment is usually hampered by the lack of reliable data on disease incidence, or treatment and control measures, and that is particularly true for swine production diseases, such as infections caused by *Streptococcus suis*. Therefore, we deployed a questionnaire survey of clinical swine veterinarians to obtain the input data needed for a stochastic model to calculate the costs caused by *S. suis*, which was implemented in three of the main swine producing countries in Europe: Germany, the Netherlands and Spain. *S. suis*-associated disease is endemic in those countries in all production phases, though nursery was the phase most severely impacted. In affected nursery units, between 3.3 and 4.0% of pigs had *S. suis*-associated disease and the mortalities ranged from 0.5 to 0.9%. In Germany, the average cost of *S. suis* per pig (summed across all production phases) was 1.30 euros (90% CI: 0.53–2.28), in the Netherlands 0.96 euros (90% CI: 0.27–1.54), and in Spain 0.60 euros (90% CI: 0.29–0.96). In Germany, that cost was essentially influenced by the expenditure in early metaphylaxis in nursery and in autogenous vaccines in sows and nursery pigs; in the Netherlands, by expenditure on autogenous vaccines in sows and nursery pigs; and in Spain, by the expenditures in early metaphylaxis and to a lesser extent by the mortality in nursery pigs. Therefore, the differences in costs between countries can be explained to a great extent by the measures to control *S. suis* implemented in each country. In Spain and in Germany, use of antimicrobials, predominantly beta-lactams, is still crucial for the control of the disease.

## Introduction

*Streptococcus suis* is an encapsulated Gram-positive bacterium naturally present in the upper respiratory tract of healthy pigs, mainly in saliva, tonsils and nasal cavities (1). Pigs are usually colonized by more than one serotype, but only a few virulent strains are responsible for the disease (2). *S. suis* can cause disease in suckling piglets and fattening pigs, but most frequently in nursery pigs. The most common clinical signs are meningitis, polyarthritis and acute death (3). *S. suis* is also a zoonotic agent that may cause severe disease in humans, characterized by meningitis, but also sepsis, arthritis or endocarditis (4). Human *S. suis* infections were considered rare in the past, but the number of cases reported has increased considerably in recent years (2).

For the last 30 years, *S. suis* infections have been considered a major problem in the swine industry worldwide, in particular in intensive pig production systems (5). *S. suis* is among the pathogens for which scientific interest has increased faster in recent years, and it is currently included among the top ten swine pathogens worldwide (6). Despite this, estimations of its economic impact are lacking. *S. suis* belongs to the group of pathogens that cause production diseases (i.e., diseases not notifiable, but with significant negative impacts on mortality, morbidity, reproduction or growth), and which include for example Porcine Reproductive and Respiratory Syndrome virus (PRRSv) (6). As for production diseases reporting is not required by law, data on their frequency of infection in farms are seldom recorded, or if recorded, results are not comparable due to the absence of a common case definition. Another problem is the lack of documented information on the costs associated to the disease (e.g., treatments). Therefore, novel approaches need to be developed for the estimation of the economic impact of swine production diseases. In data-scarce situations, such as in countries with inadequate disease surveillance infrastructures or in species for which reporting is not compulsory, questionnaire-based surveys, collecting the information directly from the people able to provide the data, may be the only alternative. Examples of the use of this methodology include the estimation of the incidence of foot-and-mouth disease in Asia, Africa and South America (7) or the incidence of leishmaniosis in dogs from south-eastern Spain (8).

An added difficulty in the case of *S. suis* infections is that presumptive diagnosis is often based on clinical signs without laboratory confirmation, although other diseases (e.g., *Glaesserella parasuis* infections) may give a similar clinical picture (3). A further complication for measuring the real impact of *S. suis* infections is that, in order to control the disease, a wide range of antimicrobial agents are sometimes used in farms both prophylactically and metaphylactically (9, 10). Despite this antimicrobial use (AMU), some animals become diseased and the prognosis of these animals is often poor (10). Moreover, widespread use of antimicrobials may result in the emergence of resistances (11). As a result, the AMU is increasingly being restricted, which has contributed a 34.6% decrease in the sales of antimicrobial agents in the 25 reporting EU countries between 2011 and 2018 (12). Pressure to reduce AMU in livestock hinders the control of *S. suis* (13), and further restrictions in AMU in coming years may result in an increase of the morbidity due to *S. suis* if not compensated by other measures.

The main objectives of this study were to estimate the frequency of disease associated with the presence of *S. suis* infections in pig farms, as well as quantify the main costs associated with the disease in three of the main pig-producing countries of Europe: Germany, the Netherlands and Spain. Such baseline information is essential to detect changes in the patterns (e.g., an increase of incidence) of *S. suis*-associated disease, to make sensible decisions on whether to allocate resources for their control, or to evaluate the efficacy of possible interventions. In order to fulfill those objectives, questionnaire-based surveys of clinical swine veterinarians were carried out to obtain input data, that were later fed to mathematical models for the calculation of the costs of disease. To allow the incorporation of variability and/or uncertainty associated with many of its inputs, a stochastic model was developed. Models for the calculation of the cost of animal diseases are commonly stochastic [e.g., (14–16)].

## Materials and Methods

### Selection of Study Areas

In order to estimate the frequency and costs of *S. suis* infections in Europe, the main pig-producing areas in three of the countries with the largest pig populations within the European Union (EU), namely Germany, the Netherlands and Spain, were selected. Germany had the largest pig production of the EU in 2019 with 22.5% of the total pigs produced (17). Within Germany, most of the data was obtained from Lower Saxony, the region with the highest pig density (18). Spain had the second largest number of pigs produced in the EU in 2019 with 21.6% of the total production (17). Within Spain, the areas selected were Aragon and Catalonia, which represented 51.6% of the total pig population in Spain, according to the Spanish agricultural census (19). Finally, the Netherlands had the sixth largest pig production of the EU in 2019, 6.8% (17), and has one of the highest density of pigs in the continent.

### Questionnaires for *S. suis*-Disease

The majority of the data needed for the model was obtained through a comprehensive questionnaire, which was administered (throughout 2019) to a group of swine clinical veterinarians aimed to be representative of the different types of pig production present in the areas of study. An initial version of the questionnaire was drafted by a panel of experts, then tested with several clinical veterinarians, and deficiencies were corrected (a copy of the final questionnaire is included in the Supplementary Material). To allow the veterinarians to collect the data requested from all the farms for which they had information, questionnaires were sent a few days in advance, and then the interview was carried out by phone to facilitate clarification of any questions. Because of the complexity of the questionnaire, the final interview took about 1 h. In total, 12 clinical veterinarians were interviewed in Spain, 10 in Germany and 11 in the Netherlands. To avoid confidentiality issues, the names of the veterinarians and the companies they worked for were not recorded.

The questionnaire for veterinarians included questions in relation to several parameters:

a) number of farms of the different types (e.g., farrowing, finishing or farrow to finish) of which they were in charge, as well as the mean number of animals of the different types within them.
b) for each production phase (i.e., suckling piglets, nursery pigs and fatteners) in those farms, the proportion of times in the last year those phases were affected by *S. suis* clinical disease, the proportion of batches affected within those phases, the proportion of animals affected within those batches, and the proportion of deaths. Because diagnosis of *S. suis* infection is usually based on clinical signs without laboratory confirmation, our case definition for the questionnaires was based on the presence of signs compatible with *S. suis* infection (i.e., arthritis, incoordination or paddling). A case-farm was a farm with at least one animal with clinical disease caused by *S. suis* infection in the last 12 months.
c) classes of the antimicrobial products, route of administration and duration of treatments in each production phase. Three types of treatments were considered: early metaphylactic, late metaphylactic and therapeutic. The term early metaphylaxis referred to the administration of antimicrobials to healthy animals in farms endemically affected by *S. suis* disease; late metaphylaxis was when the treatment was applied also to healthy animals, but there were already sick animals in the group; and therapeutic was the treatment of only sick animals.
d) proportion of farms in which autogenous vaccines were applied.
e) proportion of farms in which samples from suspected cases of *S. suis* disease were sent to a laboratory for confirmation, and proportion of times those suspected cases were actually confirmed.

The reason for requesting information independently for each production phase was that several parameters (e.g., prevalence or treatments) varied significantly between phases. Therefore, throughout the text we use the terms production phases to refer to the phases of suckling piglets, nursery pigs and fatteners; and the term production units to refer to the sites where those phases took place.

Within each country, we wanted to account for the fact that the veterinarians providing information on more farms should have more weight, but at the same time, we wanted to avoid the parameters being essentially determined just by a few veterinarians with the most farms. Therefore, we restricted the weights of the veterinarians to between 1 and 20% depending on the number of farms they provided information for (see supplementary data 2 in Supplementary Material for a detailed explanation of the calculation of weights).

Questionnaires were completed in Excel, then data extraction was implemented within the R environment version 4.0.2 (20).

### Quantifying the Costs Associated With *S. suis* Infection

**Figure 1:**
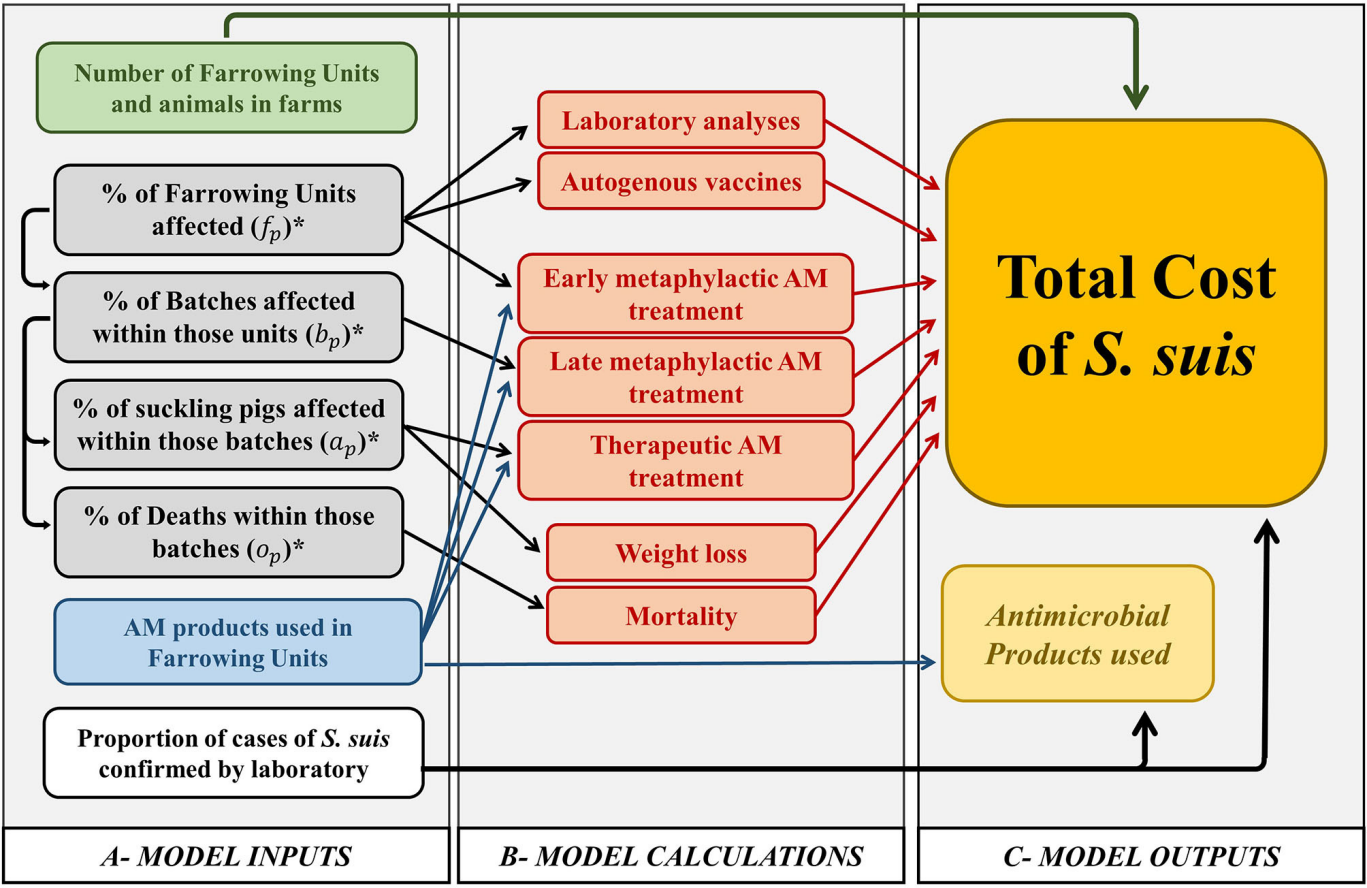
Diagram of the quantification of costs associated with *S. suis* infection and antimicrobial use in farrowing units. Gray area represents data obtained from the questionnaire for veterinarians. Red area represents estimates of the different costs associated with *S. suis* infection. Yellow area represents the outputs of calculations. *Subindex *p* refers to suckling piglets (i.e., farrowing units), similar calculations were carried out for nursery pigs in weaning units (subindex *n*) and fatteners in fattening units (subindex *f*), which when summed result in the calculation of the total cost at the end of the production cycle.

Based on the methodological framework proposed by Rushton (21), the cost of disease was the sum of the losses caused directly by the disease, and the expenditures as a result of responding to the disease. For quantifying the cost of *S. suis*-associated disease, only visible losses caused by weight loss and mortality were included (Figure 1); invisible losses such as public health costs, were not quantified. On the other hand, expenditures comprised additional costs as a result of antimicrobial treatments (early metaphylactic, late metaphylactic and therapeutic), and the expenditure on autogenous vaccines and on laboratory analyses (Figure 1). The expense of revenue forgone when denied access to better markets (21) for example, was not considered.

In order to capture the different sources of variability and uncertainty associated with the data on *S. suis*, a stochastic model was developed. The model was built so that each of the losses and expenditures considered was defined by a probability distribution. In particular, discrete distributions were used to incorporate the weights of the different questionnaires/veterinarians (22).

Given the differences between production phases, the costs of the disease were calculated independently for suckling piglets, nursery pigs and fatteners. Sub-index *p* was used for suckling piglets in farrowing units, *n* for nursery pigs in nursery units, and *f* for fatteners in fattening units. Besides, the costs of *S. suis* were calculated separately for Germany, the Netherlands and Spain, since for example the measures to control the disease and their associated costs were different.

Model calculations: First, for each country, we calculated the average costs of *S. suis* per animal (regardless of the health status) in each phase, given some level of infection in the corresponding production units (e.g., mean cost per suckling piglet in affected farrowing units in Spain). Second, the mean annual costs for each of those production units were calculated (e.g., mean annual cost per affected nursery unit in Germany). Third, the average costs per animal are summed across the three production phases to estimate total cost per pig produced in each country (e.g., mean cost of *S. suis* per pig, at the end of the production cycle, produced in the Netherlands in 2019).

#### Estimation of the Costs per Animal in Affected Production Phases

a) Losses due to weight loss
  First, for each questionnaire, we calculated the proportion of diseased suckling piglets in the farrowing units affected by *S. suis* (*d*_*p,i*_) as:
  $$\begin{array}{l} d_{p,i}=b_{p,i}\times a_{p,i}\times p_{p} \end{array}$$
  Where *b*_*p,i*_ was the proportion of batches with clinical disease in affected units according to questionnaire *i*, *a*_*p,i*_ the proportion of animals with clinical disease within those batches according to questionnaire *i*, and *p*_*p*_ was the proportion of clinical cases confirmed by the laboratory as caused by *S. suis* (Figure 1). Since the data obtained through the questionnaires was based on clinical diagnosis and there are other pathogens that may give a similar clinical picture, we had to account for that fact to obtain the real number of animals with disease caused by *S. suis*. That proportion (*p*) varied between countries and between production phases (Supplementary Table 1).
  Then, the average cost per suckling piglet due to weight loss for questionnaire *i* (*c*_*p*_*weight*_,*i*_) was calculated as:
  $$\begin{array}{l} c_{p_{weight},i}=d_{p,i}\times x_{p,i}\times v_{p} \end{array}$$
  Where *x*_*p,i*_ was the proportion of weight loss in questionnaire *i*, and *v*_*p*_ the average value of suckling piglets.
  Finally, we defined the distribution for the losses due to weight loss per suckling piglet in affected farrowing units (*c*_*p*_*weight*__) based on the weights of the different questionnaires (*w*_*p,i*_) as:
  $$\begin{array}{l} c_{p_{weight}}=Discrete\left(\left\{c_{p_{weight},i}\right\},\;\left\{w_{p,i}\right\}\right) \end{array}$$
b) Losses due to mortality
  Similarly, for each questionnaire, we calculated the proportion of suckling piglets that died in farrowing units affected by *S. suis* (*m*_*p,i*_) as Figure 1:
  $$\begin{array}{l} m_{p,i}=b_{p,i}\times o_{p,i}\times p_{p} \end{array}$$
  Where *o*_*p,i*_ was the proportion of suckling piglets that died within batches affected with *S. suis* infection in questionnaire *i*.
  Then, the average loss per suckling piglet due to mortality in affected farrowing units for questionnaire *i* (*c*_*p*_*mort,i*__) was calculated as:
  $$\begin{array}{l} c_{p_{mort,i}}=m_{p,i}\times v_{p} \end{array}$$
  And we defined the distribution for the loss per suckling piglet due to mortality in affected farrowing units (*c*_*p*_*mort*__) depending on the weights of the questionnaires as:
  $$\begin{array}{l} c_{p_{mort}}=Discrete\left(\left\{c_{p_{mort,i}}\right\},\;\left\{w_{p,i}\right\}\right) \end{array}$$
c) Expenditure on early metaphylactic antimicrobial treatment
  Different types of antimicrobials may be used as early metaphylactic treatment, so there were significant variations in the products and/or the routes of administration used, which have different costs. Therefore, for each questionnaire, and for each combination of product and route, we calculated the average expenditure per suckling piglet of that treatment (e.g., treatment number 1) (*c*_*p*_*E*1,*i*__) as:
  $$\begin{array}{l} c_{p_{E1,i}}=g_{p_{E1,i}}\times r_{p_{E1}}\times t_{p_{E1,i}}\times p_{p} \end{array}$$
  Where *g*_*p*_*E*1,*i*__ was the mean proportion of affected farrowing units in which early metaphylactic treatment number 1 (i.e., *E*_1_) was applied according to questionnaire *i*, *r*_*pE*1_ was the daily cost of that treatment per suckling piglet, *t*_*p*_*E*1,*i*__ the number of days of application according to questionnaire *i*, and *p*_*p*_ was the proportion of clinical cases confirmed. The data on the costs of the different antimicrobial treatments used in each of the countries were obtained from clinical swine veterinarians. Given the variation in prices depending on factors such as the brand or the quantity bought, an average cost was calculated for each antimicrobial and each route of administration for each country (Supplementary Table 1).
  Then, we added the different early metaphylactic antimicrobial treatments (represented by sub-index *j*) to obtain the total expenditure per suckling piglet in farrowing units according to questionnaire *i* (*c*_*p*_*earlymeta,i*__) as:
  $$\begin{array}{l} c_{p_{earlymeta,i}}=\overset{n}{\underset{j=1}{\sum }}c_{p_{Ej,i}} \end{array}$$
  Finally, we defined the distribution for the total expenditure in early metaphylactic antimicrobial treatments per suckling piglet in farrowing units (*c*_*p*_*earlymeta*__) depending on the weights as:
  $$\begin{array}{l} c_{p_{earlymeta}}=Discrete\left(\left\{c_{p_{earlymeta,i}}\right\},\;\left\{w_{p,i}\right\}\right) \end{array}$$
  Similarly, for each questionnaire, and for each combination of product and route, we calculated the average expenditure of late metaphylactic treatment number 1 per suckling piglet (*c*_*p*_*L*1,*i*__) as:
  $$\begin{array}{l} c_{p_{L1,i}}=g_{p_{L1,i}}\times r_{p_{L1}}\times t_{p_{L1,i}}\times p_{p} \end{array}$$
  Where *g*_*p*_*L*1,*i*__ was the mean proportion of affected farrowing units in which late metaphylactic treatment number 1 (i.e., *L*_1_) was applied according to questionnaire *i*, *r*_*pL*1_ was the daily cost of that treatment per suckling piglet, *t*_*p*_*L*1,*i*__ the number of days of application according to questionnaire *i*, and *p*_*p*_ was the proportion of clinical cases confirmed.
  And the same for therapeutic treatment number 1 per suckling piglet (*c*_*p*_*L*1,*i*__):
  $$\begin{array}{l} c_{p_{T1,i}}=g_{p_{T1,i}}\times r_{p_{T1}}\times t_{p_{T1,i}}\times p_{p} \end{array}$$
  Where *g*_*p*_*T*1,*i*__ was the mean proportion of affected farrowing units in which therapeutic treatment number 1 (i.e., *T*_1_) was applied according to questionnaire *i*, *r*_*pT*1_ was the daily cost of that treatment per suckling piglet, *t*_*p*_*T*1,*i*__ the number of days of application according to questionnaire *i*, and *p*_*p*_ was the proportion of clinical cases confirmed.
  Then, we added the different late metaphylactic antimicrobial treatments and the different therapeutic antimicrobial treatments. Finally, we defined the distribution for the total expenditure in late metaphylactic and therapeutic antimicrobial treatments per suckling piglet in farrowing units (*c*_*p*_*latemeta*__ and *c*_*p*_*therapeutic*__, respectively) depending on the weights.
d) Expenditure on autogenous vaccines
  First, for each questionnaire, we calculated the average expenditure per suckling piglet due to the use of autogenous vaccines (*c*_*p*_*autovac,i*__) as:
  $$\begin{array}{l} c_{p_{autovac,i}}=h_{p,i}\times k_{p} \end{array}$$
  Where *h*_*p,i*_ was the proportion of affected farrowing farms in which autogenous vaccines were used according to questionnaire *i*, and *k*_*p*_ was the vaccination cost per animal. In farrowing units, passive immunization of suckling piglets relies on the vaccination of sows, although for simplification purposes, the costs were recalculated per piglet.
  Then, we defined the distribution for the total expenditure on autogenous vaccines per suckling piglet (*c*_*p*_*autovac*__) depending on the weights as:
  $$\begin{array}{l} c_{p_{autovac}}=Discrete\left(\left\{c_{p_{autovac,i}}\right\},\;\left\{w_{p,i}\right\}\right) \end{array}$$
e) Expenditure on analyses
  First, for questionnaire *i*, we calculated the average expenditure on analyses per suckling piglet (*c*_*p*_*analyses,i*__) as:
  $$\begin{array}{l} c_{p_{analyses,i}}=l_{p,i}\times \left(\frac{s}{N_{p,i}}\;\right) \end{array}$$
  Where, *l*_*p,i*_ was the proportion of affected farrowing farms that sent samples to the laboratory for confirmation according to questionnaire *i*, *s* was the average cost of analysis including the shipping of samples and the laboratory costs and *N*_*p,i*_ was the average number of piglets produced per year per farrowing farm according to questionnaire *i*. That way, costs per farm are transformed into costs per animal.
  Finally, we defined the distribution for the total expenditure for analyses per suckling piglet (*c*_*p*_*analyses*__) depending on the weights as:
  $$\begin{array}{l} c_{p_{analyses}}=Discrete\left(\left\{c_{p_{analyses,i}}\right\},\;\left\{w_{p,i}\right\}\right) \end{array}$$

#### Estimation of the Annual Costs per Affected Production Unit

For quantifying the annual costs in affected production units, we first had to calculate the average number of animals produced in those units per year (Supplementary data 3 in Supplementary Material). By considering that, and the different losses and expenditures per animal according to questionnaire *i*, we obtained the distributions per affected production phase per year. For example, the distribution for the total annual cost due to weight loss in suckling piglets in affected farrowing units (*C*_*p*_*weight*__) was defined as:

$$\begin{array}{l} C_{p_{weight}}=Discrete\left(\left\{c_{p_{weight},i}\times N_{p,i}\right\},\;\left\{w_{p,i}\right\}\right) \end{array}$$

Where *c*_*p*_*weight*_,*i*_ was the average cost per suckling piglet due to weight loss for questionnaire *i*, *N*_*p,i*_ was the average number of suckling piglets produced per year per farrowing unit for questionnaire *i*, and *w*_*i*_ was the weight of the questionnaire *i*. Upper case “*C*” was used for the costs per unit per year, and lower case “*c*” for the costs per animal. Similarly, the distributions for the total annual losses due to mortality (*C*_*p*_*mort*__), and total annual expenditures in early metaphylactic antimicrobial treatments (*C*_*p*_*earlymeta*__), late metaphylactic antimicrobial treatments (*C*_*p*_*latemeta*__), therapeutic antimicrobial treatments (*C*_*p*_*therapeutic*__), autogenous vaccines (*C*_*p*_*autovac*__) and analyses (*C*_*p*_*analyses*__) in affected farrowing units, were also obtained.

Finally, the total cost per affected farrowing unit per year was calculated as:

$$\begin{array}{l} C_{p_{total}}=C_{p_{weight}}+C_{p_{mort}}+C_{p_{earlymeta}}+C_{p_{latemeta}}+C_{p_{therapeutic}}\\ \;+C_{p_{autovac}}+C_{p_{analyses}} \end{array}$$

#### Estimation of the Cost per Animal by Country, Summed Across All Production Phases

Finally, in a given country, the average cost due to *S. suis* for each pig at the end of the production cycle (i.e., end of fattening), was estimated. In order to do that, first the average cost per suckling piglet (*a*_*p*_*total*__), the average cost per nursery pig (*a*_*n*_*total*__) and the average cost per fattener (*a*_*f*_*total*__) was calculated as:

$$\begin{array}{l} a_{p_{total}}=c_{p_{total}}\times f_{p}\\ a_{n_{total}}=c_{n_{total}}\times f_{n}\\ a_{f_{total}}=c_{f_{total}}\times f_{f} \end{array}$$

Where, *c*_*p*_*total*__, *c*_*n*_*total*__ and *c*_*f*_*total*__ were the average costs of *S. suis* in affected units per suckling piglet, nursery pig and fattener, respectively; and *f*_*p*_, *f*_*n*_ and *f*_*f*_ were the proportions of farrowing, nursery and fattening units affected by *S. suis*-disease, respectively.

Then, for a given country, e.g., Spain (*c*_*Spain*_), by adding the average costs for the different phases of production, the average cost due to *S. suis* for each pig at the end of the production cycle was calculated:

$$\begin{array}{l} c_{Spain}=a_{p_{total}}+a_{n_{total}}+a_{f_{total}} \end{array}$$

A numerical example of the calculation of the average cost due to *S. suis* for each pig at the end of the production cycle is included as Supplementary data 4 in Supplementary Material.

##### Sensitivity Analysis

A sensitivity analysis (SA) was used to quantify the influence of the different losses and expenditures in the different production phases on the total costs of *S. suis* per pig at the end of the production cycle by country (i.e., *c*_*Germany*_, *c*_*Netherlands*_ and *c*_*Spain*_). Rank order correlation was used as recommended by the Office International des Epizooties (OIE) (23).

##### Modeling Software

The spreadsheet model was constructed in Microsoft Excel (Microsoft® Office Professional Edition, 2013), and run for 150,000 iterations using Latin Hypercube sampling as recommended (23) in @Risk version 6.1.1 (© Palisade Corporation). Such number of iterations was selected to ensure the convergence of all output parameters considering a convergence tolerance of only 1% with a confidence level of 95% for their mean values.

#### Costs of Antimicrobials by Family

Besides considering the costs of antimicrobials by types of treatment (i.e., early metaphylactic, late metaphylactic and therapeutic), the costs of antimicrobials were also calculated by antimicrobial families. Antimicrobials were grouped in the following families: beta-lactams, cephalosporins, macrolides, sulphonamides, tetracyclines and others.

### Sources of Data

In addition to all the information obtained from the questionnaires, the model for the calculation of the costs of *S. suis* required many other input parameters, which were obtained from a variety of sources. A complete list of input parameters with their values, units and sources are detailed in Supplementary Table 1.

## Results

### Questionnaires and the Occurrence of *S. suis* Infections in the Countries of Study

The clinical veterinarians interviewed were able to provide data from 1,652 production units in Germany, 480 in the Netherlands and 1,583 in Spain (Table 1).

**Table 1:** Frequency of *S. suis* infections in the countries of study, including % of units clinically affected, % of batches clinically affected within affected units, % of animals with *S. suis* disease and mortality (%) caused by *S. suis* disease.

**Phase**	**Country**	**Total units from which data was collected**	**Mean number of animals produced per unit per year**	% of units clinically affected (*f*)^*^	% of batches clinically affected within affected units (*b*)^*^	% of animals with *S. suis* disease within affected units (*d*)^*^	Mortality (%) caused by *S. suis* disease within affected units (*m*)^*^
Suckling piglets	Germany	510	10,725	64.5%	52.9%	2.2%	0.4%
	Netherlands	157	17,614	66.7%	43.3%	1.6%	0.3%
	Spain	437	25,780	80.4%	36.1%	1.2%	0.4%
Nursery pigs	Germany	468	10,620	62.0%	64.1%	3.3%	0.5%
	Netherlands	171	16,423	68.0%	65.2%	4.0%	0.9%
	Spain	370	22,665	82.9%	66.5%	3.3%	0.7%
Fatteners	Germany	674	8,173	39.8%	19.8%	0.2%	0.0%
	Netherlands	152	9,119	58.2%	28.3%	0.3%	0.1%
	Spain	776	7,774	47.1%	31.8%	0.3%	0.1%

* *Values weighted by questionnaires*.

#### Differences Between Phases

*S. suis*-associated disease is endemic in Germany, the Netherlands and Spain in all production phases, although with differences between countries and phases (Table 1). In the three countries, the phase most severely affected was nursery with high proportions of units affected (62.0–82.9%) and batches within those units (64.1–66.5%). However, as those estimates were based on clinical diagnosis, we evaluated the proportion of suspected clinical cases confirmed by the laboratory. That proportion varied between countries and particularly between production phases. In Germany, the Netherlands and Spain, the proportions of confirmation in suckling piglets were 75, 81, and 86%, respectively; in nursery 77, 91, and 76%, respectively; and in fattening 46, 81, and 50%, respectively.

Taking the probability of confirmation into account, in affected nursery units in those countries, between 3.3 and 4.0% of nursery pigs had *S. suis*-associated disease, with a mortality between 0.5 and 0.9% (Table 1). Morbidity and mortality were lower in affected farrowing units and much lower in affected fattening units (Table 1).

#### Differences Between Countries

The proportions of animals affected by *S. suis* and the mortalities in the different phases were similar between the countries (Table 1). The main discrepancies were in the proportion of animals affected in farrowing, which ranged between 1.2% in Spain and 2.2% in Germany.

### Quantification of the Costs Associated With *S. suis* Infection

#### Cost per Animal in Affected Production Phases

The mean total costs per suckling piglet in affected farrowing units were 0.86 euros in Germany, 0.61 in the Netherlands and 0.11 in Spain. In affected nursery units, the costs were higher, 1.06 euros per nursery pig in Germany, 0.73 in the Netherlands and 0.57 in Spain. In affected fattening units, the costs were much lower, 0.22, 0.11, and 0.07 euros per fattener, respectively in the three countries. The mean values for the different types of losses and expenditures and their 90% confidence intervals (CI), are shown in Table 2. The wide CI for some of the values obtained are indicative of significant variations between the costs, even within a country and a production phase.

**Table 2:** Mean losses, expenditures and total cost per animal in affected production units (in euros) for the different production phases in the countries of study.

**Phase**	**Country**	**Mortality**	**Early Metaph**.	**Late Metaph**.	**Therap**.	**Autogenous vaccines**	**Analyses**	**Total**
Suckling piglets	Germany	0.05 (0.00–0.15)	0.15 (0.00–0.50)	0.06 (0.00–0.20)	0.01 (0.00–0.02)	0.59 (0.23–0.92)	0.01 (0.00–0.02)	0.86 (0.31–1.39)
	Netherlands	0.03 (0.00–0.06)	0.00 (0.00–0.02)	-	0.00 (0.00–0.02)	0.57 (0.00–0.91)	0.00 (0.00–0.01)	0.61 (0.03–0.96)
	Spain	0.05 (0.00–0.10)	0.02 (0.00–0.12)	0.01 (0.00–0.04)	-	0.03 (0.00–0.36)	0.00 (0.00–0.01)	0.11 (0.01–0.40)
Nursery pigs	Germany	0.24 (0.05–0.70)	0.44 (0.00–1.34)	0.17 (0.02–1.81)	0.04 (0.00–0.24)	0.17 (0.00–0.92)	0.01 (0.00–0.02)	1.06 (0.19–2.49)
	Netherlands	0.24 (0.05–0.38)	0.01 (0.00–0.04)	0.02 (0.00–0.10)	0.02 (0.00–0.07)	0.44 (0.00–0.91)	0.00 (0.00–0.01)	0.73 (0.15–1.32)
	Spain	0.19 (0.03–0.38)	0.29 (0.04–0.67)	0.05 (0.00–0.08)	0.01 (0.00–0.01)	0.04 (0.00–0.22)	0.00 (0.00–0.01)	0.57 (0.24–0.94)
Fatteners	Germany	0.02 (0.00–0.05)	0.14 (0.00–0.41)	0.05 (0.00–0.53)	0.01 (0.00–0.03)	-	0.00 (0.00–0.02)	0.22 (0.01–0.54)
	Netherlands	0.05 (0.00–0.11)	0.00 (0.00–0.02)	0.00 (0.00–0.01)	0.00 (0.00–0.01)	0.04 (0.00–0.52)	0.00 (0.00–0.01)	0.11 (0.01–0.54)
	Spain	0.04 (0.01–0.17)	0.02 (0.00–0.07)	0.01 (0.00–0.05)	-	-	-	0.07 (0.01–0.23)

*In brackets, 90% confidence interval (CI) of the corresponding cost*.

Weight losses due to *S. suis* were considered negligible in the three phases. The mortality losses per nursery pig ranged from 0.19 euros in Spain to 0.24 in Germany and the Netherlands, while values for suckling piglets and fatteners were much lower.

Substantial differences were observed between countries and phases in the expenditure of early metaphylactic treatments (Table 2). The highest expenditures were in Germany, in particular in nursery pigs (0.44 euros per animal), but also in suckling piglets (0.15 euros) and fatteners (0.14 euros). In Spain, the expenditure of early metaphylaxis was important only in nursery pigs (0.29 euros per animal), while in the Netherlands it was almost negligible in all phases. The expenditure of late metaphylactic treatments were consistently low, except for nursery pigs in Germany (0.17 euros per animal). The expenditure of therapeutic treatments was even lower (Table 2).

There were important differences in relation to the expenditure on autogenous vaccines. The costs of vaccination were high in sows (included in the costs of suckling piglets) in Germany and in the Netherlands (0.59 and 0.57 euros per piglet, respectively), and in nursery pigs in the Netherlands (0.44 euros). In contrast, in Spain, spending on autogenous vaccines was low in all phases. There were even substantial discrepancies in the expenditure on autogenous vaccines within a country (as shown by the wide CI).

Finally, the costs of laboratory analyses were almost negligible in all the countries.

#### Annual Cost per Affected Production Units

By considering the average number of animals produced per year in each type of production unit, and the associated costs per animal in affected units, we calculated the average costs per affected production unit in the three countries of study. The main economic costs occurred in affected nursery units, with an average annual cost per affected unit of 9.9 thousand euros in Germany, 11.2 in the Netherlands and 14.1 in Spain. The costs were also substantial in affected farrowing units in Germany (8.7 thousand euros per affected unit) and the Netherlands (10.2 thousand euros), and much lower in Spain. In affected fattening units, the annual costs were considerably lower. The costs (mean values and 90% CI) per affected production unit in the countries of study are shown in Supplementary Table 2.

#### Cost per Animal by Country, Summed Across All Production Phases

For a given country, taking into account the average costs per animal in affected farrowing, nursery and fattening units, and the proportions of those units affected, the average cost due to *S. suis* for each pig at the end of the production cycle was calculated. By considering the proportions of units affected, the value obtained is an average cost of *S. suis* for each of the pigs produced in the country.

**Figure 2:**
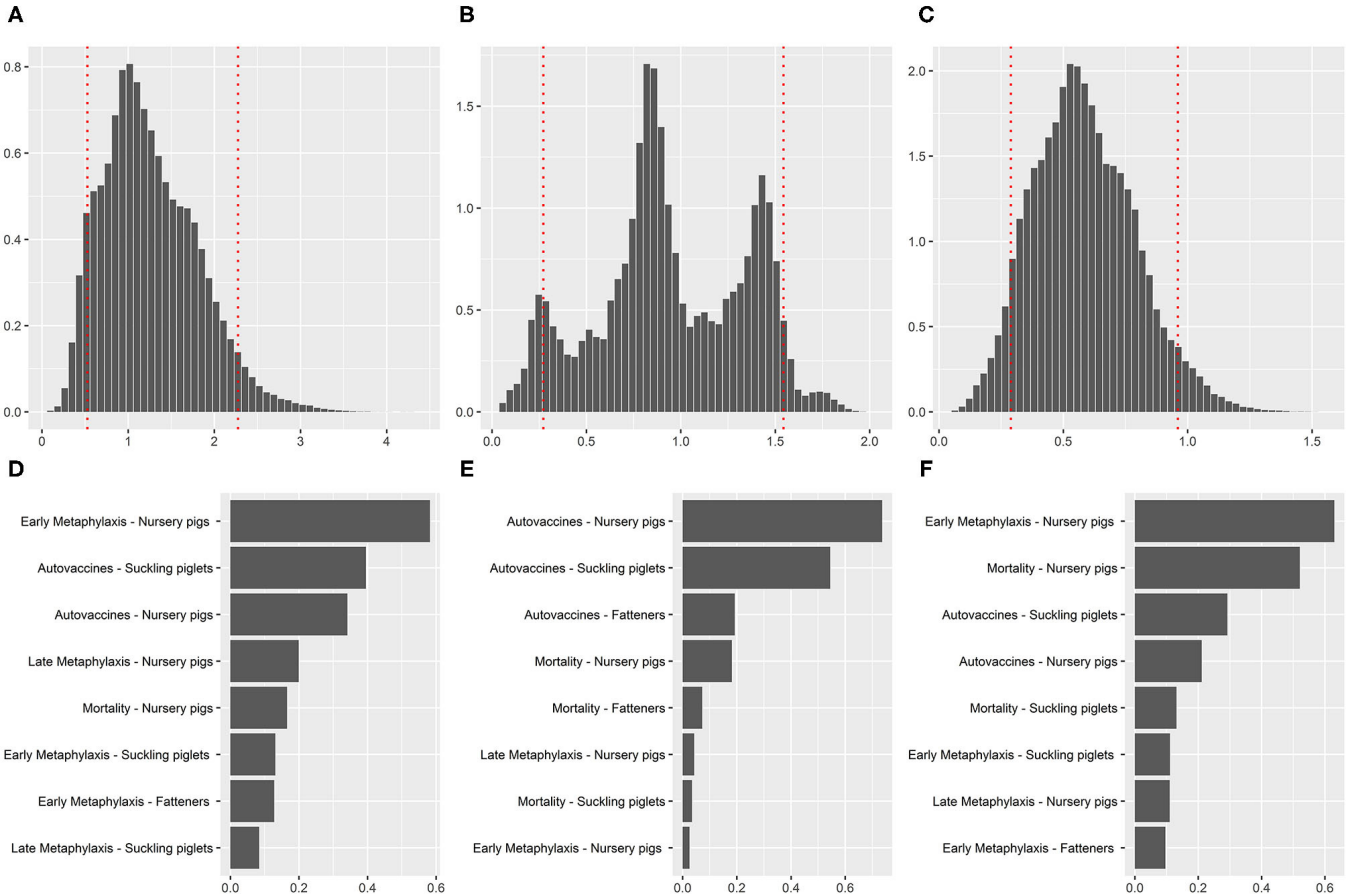
Probability distributions for the mean cost of *S. suis* (summed across all production phases) per pig obtained at the end of the production cycle in Germany **(A)**, the Netherlands **(B)** and Spain **(C)**, and results of the sensitivity analysis of those costs (by rank order correlation) in Germany **(D)**, the Netherlands **(E)** and Spain **(F)**.

In Germany, the mean cost of *S. suis* per pig at the end of the production cycle was 1.30 euros (90% CI: 0.53–2.28; in the Netherlands, 0.96 euros (90% CI: 0.27–1.54); and in Spain, 0.60 euros (90% CI: 0.29–0.96). The probability distributions for the mean cost of *S. suis* per pig at the end of the production cycle for the countries of study are shown in Figure 2. The distribution for the Netherlands had a trimodal shape, while for Germany and Spain the distributions were bell-shaped.

The sensitivity analysis (Figure 2) showed that the cost in Germany was mainly influenced by the expenditures in early metaphylaxis in nursery and in autogenous vaccines in farrowing and nursery. In the Netherlands, the expenditures on autogenous vaccines in sows and farrowing were the most influential. In Spain, the cost of *S. suis* was mainly influenced by the expenditures in early metaphylaxis and to a lesser extent by the mortality in nursery.

#### Costs of Antimicrobials by Family

**Figure 3:**
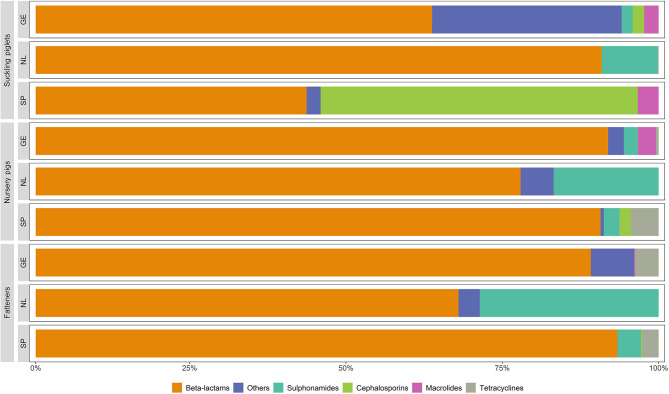
Proportions of the costs of antimicrobials per animal in affected production units by antimicrobial families for the countries of study.

The costs of antimicrobials (mean and 90% CI) per animal in affected production units in the countries of study, by antimicrobial families, are shown in Table 3. Beta-lactams represented a significant part of the cost of antimicrobials for the control of *S. suis*, in particular in nurseries in Germany and Spain (56.9 and 30.9 cents of euros per nursery pig, respectively). Cephalosporins, macrolides, sulphonamides, tetracyclines and other antimicrobials were used only occasionally in some phases and countries (Table 3; Figure 3).

**Table 3:** The costs of antimicrobials (mean and 90% CI) per animal in affected production units by antimicrobial families for the countries (cost in cents of euros).

**Phase**	**Country**	**Beta-lactams**	**Cephalosporins**	**Macrolides**	**Sulphonamides**	**Tetracyclines**	**Others**	**Total *S. suis***
Suckling piglets	Germany	13.9 (0.1–46.0)	0.4 (0.0–1.6)	0.5 (0.0–4.2)	0.4 (0.0–4.6)	<0.1 (0.0– <0.1)	6.6 (0.0–22.8)	21.7 (5.5–46.8)
	Netherlands	0.6 (0.1–2.2)	-	-	0.1 (0.0–0.2)	<0.1 (0.0– <0.1)	-	0.6 (0.1–2.2)
	Spain	1.3 (0.0–7.6)	1.5 (0.0–4.3)	0.1 (0.0–1.8)	-	-	0.1 (0.0–1.2)	2.9 (0.0–8.3)
Nursery pigs	Germany	56.9 (4.1–181.9)	<0.1 (0.0–0.2)	1.9 (0.0–29.9)	1.4 (0.0–16.0)	0.3 (0.0–4.7)	1.6 (0.0–27.3)	64.9 (4.1–181.9)
	Netherlands	3.5 (0.2–13.6)	-	-	0.8 (0.0–2.3)	-	0.2 (0.0–2.0)	4.5 (0.8–14.4)
	Spain	30.9 (8.3–53.0)	0.6 (0.0–4.8)	-	0.9 (0.0–9.4)	1.5 (0.0–20.7)	0.2 (0.0–0.9)	34.1 (8.3–56.9)
Fatteners	Germany	12.2 (0.0–101.2)	<0.1 (0.0– <0.1)	<0.1 (0.0–0.3)	-	0.5 (0.0–5.3)	1.0 (0.0–13.9)	13.7 (0.0–101.2)
	Netherlands	0.3 (0.0–1.4)	-	-	0.1 (0.0–0.7)	-	<0.1 (0.0– <0.1)	0.5 (0.0–1.4)
	Spain	2.5 (0.0–7.3)	<0.1 (0.0– <0.1)	-	<0.1 (0.0– <0.1)	<0.1 (0.0– <0.1)	<0.1 (0.0– <0.1)	2.7 (0.0–7.3)

*The value <0.1 is used for values below 0.1 but different from zero*.

## Discussion

*S. suis*-associated disease is regarded as one of the main diseases in the swine industry, in particular in intensive pig production systems (5). However, as with other production diseases such as PRRS or swine influenza, because reporting is not compulsory, there is almost no data on their occurrence, the measures by which they are currently controlled in the field, and most importantly, the losses and expenditures they cause. *S. suis*-associated disease is considered endemic in the majority of countries of the world, although studies on its frequency are lacking. Our results show that the disease is highly prevalent in German, Dutch and Spanish pig farms. The proportions of animals affected and the mortalities in the different phases were quite similar in the three countries despite differences in the proportions of units and batches affected or in the measures applied to control the disease.

There were substantial differences in the frequency of disease between phases. Nursery was the phase most frequently affected, and also where morbidity and mortality were highest, confirming previous observations (3). The mortality of *S. suis*-disease reported is usually lower than 5% (24), although in absence of treatment outbreaks could reach mortalities of 20–30% (25, 26). Decrease of maternally-derived antibodies during the nursery phase, or stress due to the movement of animals to the nursery units, or the mixing of animals from different litters, may explain why the disease is more frequent in nurseries (2, 27, 28).

Our study showed that *S. suis* also causes losses in suckling piglets, as indicated by the morbidity and mortality reported by the veterinarians we surveyed. In some farms the amount of colostrum ingested by piglets may not be adequate, which may compromise the passive maternal immunity in piglets. *S. suis*-associated disease may also occur in suckling piglets from gilts due to lower levels of antibodies (3). In contrast, *S. suis*-disease was much less of a problem for fatteners, which coincides with previous knowledge that *S. suis*-disease rarely occurred in pigs 10 weeks of age or older (29). It is believed that older animals are resistant to the disease due to the presence of high levels of antibodies (3, 28).

The causes of *S. suis*-associated disease endemicity in Germany, the Netherlands and Spain are not well-known. Intensification of pig production resulted in a shift of the relative importance of swine pathogens, with parasites becoming less common and bacterial diseases more frequent (with *S. suis* among the group that has increased faster) (6). Coinfection with viruses such as PRRSv or swine influenza virus, which are prevalent in the countries of study, results in a higher incidence of *S. suis*-disease and more severe lesions (30, 31).

As diagnosis of *S. suis* infection in the field is essentially clinical, but there are other pathogens that may give similar signs, we obtained information on the proportion of suspected cases that were actually confirmed by the laboratory. According to our results, the majority (>75%) of the clinical cases in suckling piglets and nursery pigs were confirmed, while in fattening the proportion was lower.

*S. suis* has important consequences for swine production, although with differences between the countries of study. In Germany, *S. suis*-disease primarily affected suckling piglets and nursery pigs, and to a lesser extent fatteners; in the Netherlands it largely affected suckling piglets and nursery pigs; while in Spain, *S. suis*-disease affected mainly nursery pigs. Direct losses were almost exclusively caused by mortality, as weight loss in affected animals was considered insignificant because they recovered and regained their normal weight before the end of the production cycle. The costs due to mortality were relatively similar between countries, but there were differences between phases, with much higher losses in nursery than in suckling piglets (where the mortality and the values of the animals were lower) and fattening (where the values of the animals was higher, but the mortality much lower). In Spain, mortality in nursery pigs was particularly influential on the total cost per pig at the end of the production cycle (as shown in the SA).

Antimicrobial costs of *S. suis* were considerable in Germany and Spain as a result of early metaphlyactic treatment (also revealed in the SA). Use of antimicrobials in swine production has traditionally been considered a cost-effective tool to control diseases (32); some farmers have the perception that they contribute to increased profits (33).

The pattern of AMU in each country was likely dependent on how restrictive the national legislation was in that regard. In Germany, AMU is only justified if confirmed by laboratory diagnosis or if there are epidemiological evidences it is caused by a specific pathogen. Late metaphylactic treatment is allowed, while early metaphylaxis is only justifiable in exceptional cases. In the Netherlands, early metaphylactic treatment is forbidden but late metaphylaxis is allowed, and there are further restrictions in relation to the types of antimicrobials that can be used on animals. That was reflected in our results on the expenditure in antimicrobials in the Netherlands, which was extremely low. In contrast, in Spain early metaphylaxis is not forbidden by law (34). However, since the introduction of the electronic prescription in 2019, and the new EU regulation on medicated feed, justifying this preventive treatment has become very difficult, and a reduction on AMU is likely to occur in the following years. In fact, Spain has reduced 45% the sales of antimicrobials for food-producing animals from 2014 to 2018 (12). Widespread AMU in Spain was probably influenced also by the lower prices compared to Germany and the Netherlands.

In contrast, the unit cost of autogenous vaccines was similar in the three countries, and therefore the differences in expenditure on autovaccines were determined by how often they were applied. Frequency of use was highest in the Netherlands, then in Germany, and lowest in Spain (data not shown). In fact, the SA indicated that expenditure on autogenous vaccines in farrowing and in nursery were highly influential on the total cost per pig at the end of the production cycle in Germany and the Netherlands. In the Netherlands, two very distinctive patterns in the use of autogenous vaccines occurred in the country, with many farms spending very little and many others spending quite a lot, and that was responsible for the trimodal profile of the distribution in the SA. The use of autogenous vaccines is still controversial due to the limited scientific evidence validating their efficacy and their contradictory results. Failure of autogenous vaccines has been attributed to loss of antigenicity because of the killing of the bacteria, failure in the diagnosis or selection of the strain included, or even differences between laboratories in the production process (29, 35).

The estimation of the annual cost of *S. suis* per affected units evidenced that the disease represents an important burden for pig production, although with substantial differences not only between countries, but also within countries. The mean annual costs for farmers ranged between 8.7 and 14.1 thousand euros per affected farrowing or nursery unit (with the exception of farrowing units in Spain), while costs in affected fattening units were much lower.

Considering the mean costs per animal summed across all production phases and that millions of pigs are annually produced in Germany, the Netherlands and Spain, *S. suis* causes millions of euros of annual losses to the swine sectors in those countries. However, even though the regions included in the study represent an important proportion of the pig production in the three countries, and that in the remaining regions the majority of pig sector is similarly composed (mainly of highly specialized large farms) (36), the extrapolation of results to the whole of each country may be questionable.

Previous attempts to estimate the losses associated with *S. suis*-disease have highlighted the difficulties due to data limitations. Because of the lack of incidence data, the estimate for the annual losses of *S. suis* type II in Great Britain in 1996, as calculated by Bennett et al. (37), was extremely wide, between 25 thousand and 2 million pounds. In later work, the cost ranged between 100 thousand and 1.3 million pounds (38). A high burden of *S. suis* is in agreement with a study by VanderWaal and Deen (6) that reported *S. suis* as one of the most important pathogens for the swine industry nowadays on the basis of the number of publications. Considering the (21) framework, only visible losses caused by mortality and expenditures on antimicrobial treatments, autogenous vaccines and laboratory analyses were considered. Yet, *S. suis* has other major negative consequences, such as the impact of *S. suis* as an emerging zoonotic agent, which has increased in the last 15–20 years (3). However, because of the lack of incidence data, the costs of human infections could not be included in our study. The only study in which the human cost of *S. suis* was calculated was carried out in Vietnam by Huong et al. (39), who estimated that the annual cost was between 2.64 and 3.38 million US$.

Another unaccounted effect of *S. suis* is the potential for AMU to control the disease increasing the risk for development of antimicrobial resistance (13). In fact, there are growing evidences of the occurrence of antimicrobial resistance in *S. suis* (9, 40, 41). In order to reduce AMU, new EU regulations to be implemented in 2022 include restrictions on the prophylactic and metaphylactic antimicrobial treatment of animals (42, 43). In this context of a progressive reduction of AMU, development of effective alternative tools (e.g., vaccines), is essential to control *S. suis* (29). In the absence of such tools, good biosecurity, plus management practices (e.g., all-in/all-out, groups with similar ages, improvement of ventilation, or avoiding overcrowding) are key for the control of *S. suis* (9). Also (44) suggest that some feed additives could be useful to help reduce the impact of *S. suis*-associated disease.

While (45) found significant differences between countries in the patterns of the antimicrobial families used, that was not observed for the control *S. suis* in Germany, the Netherlands and Spain, which relied almost exclusively on the use beta-lactams in all the phases. Still, use of cephalosporins was reported in Germany and Spain, an antimicrobial family classified as critically important for human health. Differences in the families of antimicrobials used could be related to differences in market prices, driven by veterinarians' own experiences or country regulations, as in the case of cephalosporins, forbidden in the Netherlands for food producing animals (46, 47).

Our study has several limitations that need to be taken into account. Given the complexity of the questionnaire, and to avoid non-response bias, the veterinarians had to be selected by convenience among known clinical veterinarians. Even though the sample was meant to be representative of the different types of pig production present in the areas of study, some sort of selection bias cannot be ruled out. Also, our results rely on the accuracy with which all the data requested in the questionnaire was remembered and reported by the veterinarians. While some recall bias is likely, we consider that the parameters estimated were a good approximation of the real values. To improve the precision of the data, the questionnaires were sent a few days in advance to allow the veterinarians to collect the data requested, and then the questionnaire was filled out by phone interview to facilitate the clarification of any possible doubt. Telephone interviews share many of the advantages of face-to-face interviews (e.g., high response rate, opportunity to explain the study) but are less time consuming and less expensive (48). Furthermore, there are some extra costs associated with *S. suis* in animals that die throughout the production cycle, as for example those animals may have received early metaphylactic treatment or autovaccines for *S. suis* before they died. However, considering that they apply only to some of the costs calculated before and that mortalities in the countries of study are generally low, its impact is likely to be limited. A similar extra costs is incurred by the pigs that do not complete the production cycle because they are slaughtered at earlier stages (e.g., suckling piglets), although its economic impact is also likely to be restricted. For simplification purposes, those extra costs were not considered in the calculations of the cost.

The economic assessment of animal diseases is often hampered by the lack of reliable data, and that is particularly true for swine production diseases. The evaluation of the cost of a disease relies on the availability of three main types of information: the incidence of the disease, how the disease is distributed among the population, and the treatment and control measures (37). That kind of data is essential for detecting changes in the incidence or prevalence of the disease, deciding whether control measures are needed, or evaluating the implementation of those measures. While technological progress has contributed to the development of tools that allow monitoring the occurrence of endemic diseases in almost real-time, e.g., Alba-Casals et al. (49), their application is still restricted to a limited number of farms/companies, which are not necessarily representative of the whole swine sector. Therefore, alternative methods need to be used for the assessment of endemic diseases and their impact at the country level. We combined questionnaire-based surveys of clinical swine veterinarians with mathematical models. Questionnaires allowed us to collect data on many parameters related to *S. suis*-disease, from a very large number of farms, with a minimum cost; a strategy that may be easily adapted to other production diseases. Bennett and IJpelaar (38) also relied on surveys, in that case of experts, to obtain the input data needed for the economic evaluation of several livestock diseases. The use of a stochastic model allows both the variability as well as the uncertainty associated with the data on *S. suis* to be incorporated into the calculations. In veterinary medicine, stochastic models have been commonly applied to quantify the risk of introducing a disease into a country through the importation of animals or their products (22, 50), but are increasingly being used for the calculation of the cost of diseases [e.g., (14–16)].

## Data Availability Statement

The original contributions presented in the study are included in the article/Supplementary Material, further inquiries can be directed to the corresponding author.

## Author Contributions

CN-I, JC, and SN conceived the study. CN-I, JC, IH-P, NS-Z, LP-G, and SN contributed to the acquisition of data or its analysis. CN-I and SN drafted the first version of the article. CN-I, JC, IH-P, NS-Z, MG, LM-G, and SN revised critically the article. All authors approved of the version submitted.

## Conflict of Interest

The authors declare that the research was conducted in the absence of any commercial or financial relationships that could be construed as a potential conflict of interest.

## Publisher's Note

All claims expressed in this article are solely those of the authors and do not necessarily represent those of their affiliated organizations, or those of the publisher, the editors and the reviewers. Any product that may be evaluated in this article, or claim that may be made by its manufacturer, is not guaranteed or endorsed by the publisher.
