# Supplementary material for: Stochastic Assessment of the Economic Impact of Streptococcus suis-Associated Disease in German, Dutch and Spanish Swine Farms
Source: Front Vet Sci. 2021 Aug 19;8:676002. doi: 10.3389/fvets.2021.676002 (PMC8417327; doi:10.3389/fvets.2021.676002)
Supplement: Supplementary file 1 [file Table_1.DOCX]

# SUPPLEMENTARY TABLE 1

Supplementary table 1: Values used for the parameters in the study in the different countries. *Values were calculated 3 days before movement to a nursery unit. ^+^ Values were calculated in the middle of the nursery period. ^±^ Values were calculated 5 days after movement to fattening unit.

| **Parameter** | **Abbreviation** | **German values** | **Dutch values** | **Spanish values** | **Unit** | **Source of data** |
| --- | --- | --- | --- | --- | --- | --- |
| **Proportion of weight loss in suckling piglets** | $w_{p}$ | ≈0 | ≈0 | ≈0 | - | Questionnaire / Expert opinion |
| **Average value of a suckling piglet *** | $v_{p}$ | 13.6 | 9.5 | 11.7 | Euros | (17) / Expert opinion |
| **Proportion of weight loss in nursery pigs** | $w_{n}$ | ≈0 | ≈0 | ≈0 | - | Questionnaire / Expert opinion |
| **Average value of a nursery pig ^+^** | $v_{n}$ | 45.6 | 28.1 | 27.0 | Euros | (17) / Expert opinion |
| **Proportion of weight loss in fatteners** | $w_{f}$ | ≈0 | ≈0 | ≈0 | - | Questionnaire / Expert opinion |
| **Average value of a fattener ^±^** | $v_{f}$ | 79.1 | 46.3 | 45.1 | Euros | (17) / Expert opinion |
| **Average number of sows in a year in farrowing units** | $S$ | 2689 | 2886 | 4768 | Sows | Questionnaire |
| **Average number of piglets weaned per sow per year** | $y_{p}$ | 30.2 | 30.6 | 27.5 | Piglets/year | (51) |
| **Average total mortality during nursery** | $m_{n}$ | 2.9% | 2.8% | 4.6% | - | (51) |
| **Average number of cycles per year in nursery** | $c_{n}$ | 6.3 | 6.8 | 6.8 | Cycles/year | Expert opinion |
| **Average number of fatteners produced in a year in fattening units** | $N_{f}$ | 19225 | 15957 | 19704 | Fatteners | Questionnaire |
| **Average cost of autogenous vaccines per animal** | $k$ | 0.9 | 0.9 | 0.9 | Euros | Expert opinion |
| **Proportion of clinical cases confirmed by the laboratory in suckling piglets** | $p_{p}$ | 75.0% | 81.0% | 86.0% | - | Questionnaire |
| **Proportion of clinical cases confirmed by the laboratory in nursery pigs** | $p_{n}$ | 77.2% | 91.0% | 76.0% | - | Questionnaire |
| **Proportion of clinical cases confirmed by the laboratory in fattening** | $p_{f}$ | 45.8% | 80.6% | 50.0% | - | Questionnaire |
| **Average cost of laboratory analysis per farm** | $s$ | 64.0 | 79.2 | 33.2 | Euros | Expert opinion |
| **Average animal weight in suckling piglets *** | - | 5.5 | 5.7 | 5.4 | Kg | Expert opinion |
| **Average animal weight in nursery pigs ^+^** | - | 18.4 | 16.9 | 12.5 | Kg | Expert opinion |
| **Average animal weight in fattening ^±^** | - | 31.9 | 27.9 | 20.9 | Kg | Expert opinion |
| **Average animal price in 2019** | - | 2.48 | 1.66 | 2.16 | €/kg | (17) |
| **Pigs produced in 2019** | - | 55.1 | 16.6 | 53.0 | Millions of heads | (17) |
| **Amoxicillin – Parenteral** | - | 1.6 | 1.4 | 0.5 | Cent €/ kg of live weight | Expert opinion |
| **Amoxicillin – Premix** | - | 0.2 | 0.3 | 0.2 | Cent €/ kg of live weight | Expert opinion |
| **Amoxicillin – Oral powder** | - | 0.3 | 0.3 | 0.2 | Cent €/ kg of live weight | Expert opinion |
| **Ceftiofur – Parenteral** | - | 1.9 | - | 0.9 | Cent €/ kg of live weight | Expert opinion |
| **Penicillin – Parenteral** | - | 1.1 | 0.7 | 0.5 | Cent €/ kg of live weight | Expert opinion |
| **Trimethoprim sulfamethoxazole – Parenteral** | - | 0.7 | 0.6 | - | Cent €/ kg of live weight | Expert opinion |
| **Trimethoprim sulfamethoxazole – Premix** | - | 0.2 | 0.2 | - | Cent €/ kg of live weight | Expert opinion |
| **Trimethoprim sulfamethoxazole – Oral powder** | - | 0.3 | 0.2 | - | Cent €/ kg of live weight | Expert opinion |
