# Supplementary material for: Stochastic Assessment of the Economic Impact of Streptococcus suis-Associated Disease in German, Dutch and Spanish Swine Farms
Source: Front Vet Sci. 2021 Aug 19;8:676002. doi: 10.3389/fvets.2021.676002 (PMC8417327; doi:10.3389/fvets.2021.676002)
Supplement: Supplementary file 2 [file Table_2.DOCX]

# SUPPLEMENTARY TABLE 2

Supplementary table 2: Mean total annual cost per affected production unit in the countries of study (in thousands of euros). In brackets, 90% confidence interval (CI) of the corresponding cost.

| **Phase** | **Country** | **Mean total cost (90% CI) in thousands of euros** |
| --- | --- | --- |
| **Suckling piglets** | **Germany** | 8.7 (3.0-16.7) |
|  | **Netherlands** | 10.2 (0.5-22.9) |
|  | **Spain** | 2.7 (0.1-8.3) |
| **Nursery pigs** | **Germany** | 9.9 (1.8-25.2) |
|  | **Netherlands** | 11.2 (2.4-23.2) |
|  | **Spain** | 14.1 (4.0-27.1) |
| **Fatteners** | **Germany** | 1.2 (0.1-3.4) |
|  | **Netherlands** | 0.8 (0.0-2.4) |
|  | **Spain** | 0.4 (0.0-1.4) |
