# Supplementary material for: Stochastic Assessment of the Economic Impact of Streptococcus suis-Associated Disease in German, Dutch and Spanish Swine Farms
Source: Front Vet Sci. 2021 Aug 19;8:676002. doi: 10.3389/fvets.2021.676002 (PMC8417327; doi:10.3389/fvets.2021.676002)
Supplement: Supplementary file 3 [file Data_Sheet_1.PDF]

PIGSs Project

Questionnaire to estimate the burden of disease caused by *Streptococcus suis* in Europe

Objective

PIGSs (Program for Innovative Global prevention of *Streptococcus suis*) is a H2020 project funded by the European Union. The goal of this project is to increase our knowledge about *S. suis* infections in pigs. Despite being considered one of the most important diseases in pig production, there is almost no information on the burden of the disease in Europe. However, farmers and veterinarians are well aware of the problems caused by of *Streptococcus suis* . Therefore, the objective of the questionnaire is to gather knowledge on the presentation of the disease from clinical vets.

All information collected will be treated confidentially, there are no questions about the identity of the company or the veterinarian.

The questionnaire has been developed by the Institute of Agrifood Research and Technology - Animal Health Research Center (IRTA-CReSA), Wageningen University Research Centre and the University of Veterinary Medicine Hannover.
More information available at: www.pigss-horizon2020.eu

* Throughout the questionnaire, we are interested on the current burden of disease caused by *S. suis* , so the **data provided must only include the last 12 months** in order to include all seasons in the study.

VETERINARIAN	
Nationality	
Province(s) where the farms are	
In your opinion, is the disease caused by *Streptococcus suis* one of the 3 main diseases in pigs nowadays? Between the 4th and 10th disease by importance? Or not within the 10 most important diseases?	

Date

Refer to the next table (table 1), we consider a farm as that site/sites that have a unique identification farm number:
Number of farms: farms of the different types that are routinely visited by the veterinarian filling up the questionnaire, not only for issues related to *S. suis* but also for other reasons (vaccination, biosecurity, other diseases ...).

Number of farms suspected: farms with at least 1 animal suspected of having suffered clinical disease caused by *S. suis* infection in the last 12 months.

Table 1.- TYPE OF FARM (SITES)	NUMBER OF FARMS	NUMBER OF FARMS SUSPECTED	Mean number of animals in suspected farms (sows in farrowing units)
Farrowing (site 1)			
Weaning (site 2)			
Finishing (site 3)			
Farrowing and weaning (1 and 2)			
Wean to finish (2 and 3)			
Farrow to finish (1, 2 and 3)			

Refer to tables 2 and 3, we consider a batch as a group of animals of the same age:
If there is more than one farm type suspected (for example weaning and finishing), in the questions that follow, fill in the cells corresponding to the suspected farm types (in this example, weaning and finishing).
If there is any farm type suspected in more than one farm (for example farrowing suspected in 3 farms), the answers must be an average value for the suspected farms (for example, average proportion of batches with disease in the farrowing units of those 3 farms).
If the suspected farms are multi-sites, in the questions that follow, fill in only the data corresponding to the phase or phases suspected (for example, in a multiphase 1 and 2, if only the farrowing unit is suspected, then only fill up the questions for phase 1).

Table 2.-In farms suspected (with at least one piglet with *S. suis* problems)	IN FARROWING (1)	IN WEANING (2)	IN FINISHING (3)
Proportion of **batches with disease** associated to *S. suis* (in suspected farms)	%	%	%
Proportion of **animals with disease** associated to *S. suis* (in suspected batches)	%	%	%
Proportion of mortality, **animals dead** due to *S. suis* (over total population in suspected batches)	%	%	%
**Have suspected batches** reduced weight gain, were wasted or had lack of uniformity? If **yes**, indicate proportion of those who did.	%	%	%

Refer to table 3, the total percentage of batches suspected of *S. suis* clinical disease by season has to be 100 % (for example, for phase 1, 40% of the suspected batches occurred in spring, 10% in summer, 30% in autumn and 20% in winter).

Table 3.- Seasonality of the batches suspected of *S. suis* clinical disease (% by season)	IN FARROWING (1)	IN WEANING (2)	IN FINISHING (3)
Spring	%	%	%
Summer	%	%	%
Autumn	%	%	%
Winter	%	%	%

Refer to table 4, choose (for each phase) the three most important causes: assign the number 1 for the most important cause, 2 for the second and 3 for the third.

Table 4.- According to your experience, what are the main risk factors associated with the disease in your farms?	IN FARROWING (1)	IN WEANING (2)	IN FINISHING (3)
Animal density			
Other concomitant diseases (PRRS, circovirus, influenza...)			
Temperature fluctuation			
Poor ventilation			
Recent movement to post-weaning / fattening units			
Failure in the antimicrobial treatment			
Others (indicate)			

Refer to table 5, the total percentage of clinical signs has to be 100% for each phase that presents the disease.

Table 5.- Among the animals with disease, proportion with the following clinical signs:	IN FARROWING (1)	IN WEANING (2)	IN FINISHING (3)
Nervous signs	%	%	%
Arthritis (lameness)	%	%	%
Acute death	%	%	%
Other (indicate which)	%	%	%

Table 6.- Related with laboratory diagnosis, **in the last year**:	IN FARROWING (1)	IN WEANING (2)	IN FINISHING (3)
Proportion of **farms** with suspected *S. suis* disease from which **samples** are sent to the laboratory for confirmation	%	%	%
Proportion of those **farms** that sent samples to the laboratory in which ***S. suis*** has been **isolated**	%	%	%

Refer to the next tables (tables 7a, 7b, 7c and 7d):
The route could be water, feed or parenteral. Fill up all the sites where the disease was present. Indicate if there is no treatment in the correspondig cell (for example, the full batch is not treated when a case of *S. suis* occurs in transition).

Table 7a.- **Routine** treatment (for **all** farms)	IN FARROWING (1)			IN WEANING (2)			IN FINISHING (3)		
	% farms	Route	Duration	% farms	Route	Duration	% farms	Route	Duration
Antibiotic 1 (Name:________________________________)	%			%			%		
Antibiotic 2 (Name:________________________________)	%			%			%		
Antibiotic 3 (Name:________________________________)	%			%			%		
Antibiotic 4 (Name:________________________________)	%			%			%		
Antibiotic 5 (Name:________________________________)	%			%			%		

Table 7b.- In case of disease, therapeutic treatment of the **animals affected**	IN FARROWING (1)			IN WEANING (2)			IN FINISHING (3)		
	% farms	Route	Duration	% farms	Route	Duration	% farms	Route	Duration
Antibiotic 1 (Name:________________________________)	%			%			%		
Antibiotic 2 (Name:________________________________)	%			%			%		
Antibiotic 3 (Name:________________________________)	%			%			%		
Antibiotic 4 (Name:________________________________)	%			%			%		
Antibiotic 5 (Name:________________________________)	%			%			%		

Table 7c.- In case of disease, therapeutic treatment of the **rest of the pen**	IN FARROWING (1)			IN WEANING (2)			IN FINISHING (3)		
	% farms	Route	Duration	% farms	Route	Duration	% farms	Route	Duration
Antibiotic 1 (Name:____)	%			%			%		
Antibiotic 2 (Name:____)	%			%			%		
Antibiotic 3 (Name:____)	%			%			%		
Antibiotic 4 (Name:____)	%			%			%		
Antibiotic 5 (Name:____)	%			%			%		

Table 7d.- In case of disease, therapeutic treatment of the **rest of the batch**	IN FARROWING (1)			IN WEANING (2)			IN FINISHING (3)		
	% farms	Route	Duration	% farms	Route	Duration	% farms	Route	Duration
Antibiotic 1 (Name:____)	%			%			%		
Antibiotic 2 (Name:____)	%			%			%		
Antibiotic 3 (Name:____)	%			%			%		
Antibiotic 4 (Name:____)	%			%			%		
Antibiotic 5 (Name:____)	%			%			%		

Refer to table 8, answer with Yes or No in the different phases in which the disease is observed. If the answer is Yes**,** indicate the percentage of farms in which autovaccines is used.

Table 8.- Use of autovaccines in the last 12 months	IN FARROWING (1)	IN WEANING (2)	IN FINISHING (3)
**Yes** / **No** (if **Yes**, % of farms)	%	%	%
In what proportion of the farms where the autovaccine was applied do you consider it was successful in preventing clinical disease compatible with *S. suis* ?	%	%	%

Thank you very much for your collaboration.
