# Supplementary material for: Stochastic Assessment of the Economic Impact of Streptococcus suis-Associated Disease in German, Dutch and Spanish Swine Farms
Source: Front Vet Sci. 2021 Aug 19;8:676002. doi: 10.3389/fvets.2021.676002 (PMC8417327; doi:10.3389/fvets.2021.676002)
Supplement: Supplementary file 4 [file Data_Sheet_2.docx]

# SUPPLEMENTARY FILE 2: CALCULATION OF WEIGHTS

Within each country, we collected the data through a questionnaire administered veterinarians, each of which provided information from a number of farms that contained a number of farrowing units with an average number of sows, a number of nursery units with an average number of nursery pigs, and a number of fattening units with an average number of fatteners. We wanted to account for the fact that those parameters from the veterinarians providing information on more units and animals should have more weight, but at the same time, we wanted to avoid the parameters being essentially determined just by a few veterinarians. Therefore, we restricted the weights of the veterinarians to up to 20% depending on the number of farms they provided information for. Those averaged weighted values were the inputs fed to the mathematical model.

First, the unadjusted weights of the questionnaires/veterinarians for suckling piglets were calculated as:

$$\boldsymbol{w}_{\boldsymbol{p,i}}\left( \boldsymbol{unadjusted} \right)\boldsymbol{=}\frac{\boldsymbol{1}}{\boldsymbol{2}}\left( \frac{\boldsymbol{u}_{\boldsymbol{p,i}}}{\sum\boldsymbol{u}_{\boldsymbol{p,i}}}\boldsymbol{+}\frac{\boldsymbol{n}_{\boldsymbol{p,i}}}{\sum\boldsymbol{n}_{\boldsymbol{p,i}}} \right)$$

Where $\boldsymbol{u}_{\boldsymbol{p,i}}$ was the number of farrowing units for which questionnaire/veterinarian $\boldsymbol{i}$ had data, and $\boldsymbol{n}_{\boldsymbol{p,i}}$ was the average number of sows in those farrowing units from questionnaire/veterinarian $\boldsymbol{i}$.

Then, those values were limited to between 1% and 20%:

If $\boldsymbol{w}_{\boldsymbol{p,i}}\boldsymbol{<0.01}$ then $\boldsymbol{w}_{\boldsymbol{p,i}}\left( \boldsymbol{limited} \right)\boldsymbol{=}\boldsymbol{0.01}$; if $\boldsymbol{w}_{\boldsymbol{p,i}}\boldsymbol{>0.2}$ then $\boldsymbol{w}_{\boldsymbol{p,i}}\left( \boldsymbol{limited} \right)\boldsymbol{=}\boldsymbol{0.2}$; otherwise $\boldsymbol{w}_{\boldsymbol{p,i}}\left( \boldsymbol{limited} \right)\boldsymbol{=}\boldsymbol{w}_{\boldsymbol{p,i}}$.

Finally, values were readjusted so that the sum of final weights equals 1:

$$\boldsymbol{w}_{\boldsymbol{p,i}}\boldsymbol{=}\frac{\boldsymbol{w}_{\boldsymbol{p,i}}\left( \boldsymbol{limited} \right)}{\sum\boldsymbol{w}_{\boldsymbol{p,i}}\left( \boldsymbol{limited} \right)}$$
