# Supplementary material for: Stochastic Assessment of the Economic Impact of Streptococcus suis-Associated Disease in German, Dutch and Spanish Swine Farms
Source: Front Vet Sci. 2021 Aug 19;8:676002. doi: 10.3389/fvets.2021.676002 (PMC8417327; doi:10.3389/fvets.2021.676002)
Supplement: Supplementary file 5 [file Data_Sheet_3.DOCX]

# SUPPLEMENTARY FILE 3: ESTIMATION OF THE NUMBER OF ANIMALS PRODUCED BY UNIT PER YEAR

In a farrowing unit, the number of animals (suckling piglets) produced per year ($\boldsymbol{N}_{\boldsymbol{p,i}}$) was estimated as:

$$\boldsymbol{N}_{\boldsymbol{p,i}}\boldsymbol{=}\boldsymbol{S}_{\boldsymbol{p,i}}\boldsymbol{\times y}$$

Where $\boldsymbol{S}_{\boldsymbol{p,i}}$ was the mean number of sows in farrowing units in questionnaire $\boldsymbol{i}$, and $\boldsymbol{y}$ was the average number of piglets weaned per sow per year. We assumed that the mortality associated to *S. suis* occurred at the end of farrowing.

As disease in nursery units may occur at any time, the mean number of nursery pigs produced per year according to questionnaire/veterinarian $\boldsymbol{i}$ ($\boldsymbol{N}_{\boldsymbol{n,i}}$) was calculated as:

$$\boldsymbol{N}_{\boldsymbol{n,i}}\boldsymbol{=}\frac{\boldsymbol{N}_{\boldsymbol{p,i}}\boldsymbol{+}\left[ \boldsymbol{N}_{\boldsymbol{p,i}}\boldsymbol{\times}\boldsymbol{(1-m}_{\boldsymbol{tn}}\boldsymbol{)} \right]}{\boldsymbol{2}}$$

Where $\boldsymbol{m}_{\boldsymbol{tn}}$ was the average total mortality during nursery due to any cause.

In fattening units, since *S. suis* affects fatteners at the beginning of the fattening period, the mean number of fatteners produced per year ($\boldsymbol{N}_{\boldsymbol{f}}$) was considered equal to the number of nursery pigs at the end of the nursery period.

In farms without a farrowing unit, the number of nursery pigs produced per year for questionnaire/veterinarian $\boldsymbol{i}$ ($\boldsymbol{N}_{\boldsymbol{n,i}}$) was estimated as:

$$\boldsymbol{N}_{\boldsymbol{n,i}}\boldsymbol{=}\boldsymbol{n}_{\boldsymbol{n,i}}\boldsymbol{\times}\boldsymbol{c}_{\boldsymbol{n}}$$

Where $\boldsymbol{n}_{\boldsymbol{n,i}}$ was the average number of nursery pigs in the nursery units in questionnaire $\boldsymbol{i}$, and $\boldsymbol{c}_{\boldsymbol{n}}$ was the average number of cycles in nursery per year. And, in a farm without a farrowing unit, the number of fattening pigs produced per year ($\boldsymbol{N}_{\boldsymbol{f,i}}$) was estimated as:

$$\boldsymbol{N}_{\boldsymbol{f,i}}\boldsymbol{=}\boldsymbol{n}_{\boldsymbol{f,i}}\boldsymbol{\times}\boldsymbol{c}_{\boldsymbol{f}}$$

Where $\boldsymbol{n}_{\boldsymbol{f}}$ was the average number of fatteners in the fattening units in questionnaire $\boldsymbol{i}$, and $\boldsymbol{c}_{\boldsymbol{f}}$ was the average number of fattening cycles per year.
