# Supplementary material for: Stochastic Assessment of the Economic Impact of Streptococcus suis-Associated Disease in German, Dutch and Spanish Swine Farms
Source: Front Vet Sci. 2021 Aug 19;8:676002. doi: 10.3389/fvets.2021.676002 (PMC8417327; doi:10.3389/fvets.2021.676002)
Supplement: Supplementary file 6 [file Data_Sheet_4.docx]

# SUPPLEMENTARY FILE 4: NUMERICAL EXAMPLE OF THE CALCULATION OF THE AVERAGE COST DUE TO *S. SUIS* FOR EACH PIG AT THE END OF THE PRODUCTION CYCLE PRODUCED IN COUNTRY X

In country X, the parameters related to *S. suis*-associated disease were:

a) the average costs of *S. suis* per suckling piglet in affected farrowing units ($c_{p_{total}}$) was 0.3 €.

b) the average costs of *S. suis* per nursery pig in affected nursery units ($c_{n_{total}}$) was 0.8 €.

c) the average costs of *S. suis* per fattener in affected fattening units ($c_{f_{total}}$) was 0.2 €.

d) the proportion of farrowing units affected by *S. suis*-disease ($f_{p}$) was 0.5 (i.e., 50%).

e) the proportion of nursery units affected by *S. suis*-disease ($f_{n}$) was 0.6.

f) the proportion of fattening units affected by *S. suis*-disease ($f_{f}$) was 0.4.

Therefore, in country X, the average cost per suckling piglet ($a_{p_{total}}$), the average cost per nursery pig ($a_{n_{total}}$) and the average cost per fattener ($a_{f_{total}}$) can be calculated as:

$$a_{p_{total}}=c_{p_{total}}\times f_{p}=0.3\times0.5=0.15 €$$

$$a_{n_{total}}=c_{n_{total}}\times f_{n}=0.8\times0.6=0.48 €$$

$$a_{f_{total}}=c_{f_{total}}\times f_{f}=0.2\times0.4=0.08 €$$

Then, the average cost due to *S. suis* for each pig at the end of the production cycle produced in country X can be calculated as:

$c_{Spain}=a_{p_{total}}+a_{n_{total}}+a_{f_{total}}$=$0.15+0.48+0.08=0.71 €$
